# Pretreatment of Small-for-Size Grafts *In Vivo* by **γ**-Aminobutyric Acid Receptor Regulation against Oxidative Stress-Induced Injury in Rat Split Orthotopic Liver Transplantation

**DOI:** 10.1155/2013/149123

**Published:** 2013-10-08

**Authors:** Tomohide Hori, Shinji Uemoto, Lindsay B. Walden, Feng Chen, Ann-Marie T. Baine, Toshiyuki Hata, Justin H. Nguyen

**Affiliations:** ^1^Department of Neuroscience, Mayo Clinic, Jacksonville, FL 32224, USA; ^2^Division of Hepato-Biliary-Pancreatic and Transplant Surgery, Department of Surgery, Kyoto University Graduate School of Medicine, 54 Shogoinkawara-cho, Sakyo-ku, Kyoto 606-8507, Japan; ^3^Division of Transplant Surgery, Department of Transplantation, Mayo Clinic, Jacksonville, FL 32224, USA

## Abstract

*Background*. Graft pretreatment to limit postoperative damage has the advantage of overcoming a current issue in liver transplantation (LT). The strategic potential of graft pretreatment *in vivo* by a specific agonist for **γ**-aminobutyric acid receptor (GABAR) was investigated in the rat LT model with a small-for-size graft (SFSG). *Methods*. Recipient rats were divided into three groups according to donor treatments and recipient surgeries: (i) saline and laparotomy, (ii) saline and split orthotopic liver transplantation (SOLT) with 40%-SFSG, and (iii) GABAR agonist and SOLT with 40%-SFSG. Survival was evaluated. Blood and liver samples were collected 6 h after surgery. Immunohistological assessment for apoptotic induction and western blotting for 4-hydroxynonenal, ataxia-telangiectasia mutated kinase (ATM), histone H2AX, phosphatidylinositol-3 kinase (PI3K), Akt, and free radical scavenging enzymes were performed. *Results*. Pretreatment by GABAR showed improvement in survival, histopathological assessment, and biochemical tests. Apoptotic induction and oxidative stress were observed after SOLT with an SFSG, and this damage was limited by GABAR regulation. GABAR regulation appeared to reduce DNA damage via the ATM/H2AX pathway and to promote cell survival via the PI3K/Akt pathway. *Conclusions*. Pretreatment *in vivo* by GABAR regulation improves graft damage after SOLT with an SFSG. This strategy may be advantageous in LT.

## 1. Introduction

Oxygen is required for cell survival. However, oxygen also poses a potential hazard via reactive oxygen species (ROS) and reactive nitrogen species (RNS), with biological and functional alterations of lipids, proteins, and deoxyribonucleic acid (DNA) [1–3]. Therefore, ROS/RNS have been initially considered as harmful products of the normal aerobic metabolism. The control of ROS/RNS production plays physiological roles, especially, in regulating cell signaling to involve cell proliferation, differentiation, and apoptosis [1–3]. Oxidative stress (OS) mediated by free radicals is defined as an imbalance between the production of ROS/RNS and the antioxidant capacity of the cell [1–3]. These antioxidants ensure a defense against ROS/RNS-induced OS [2].

The predominant inhibitory neurotransmitter in the brain is *γ*-aminobutyric acid (GABA), and almost all researchers have focused on GABA or the regulation of GABA receptor (GABAR) in the brain [4–8]. Currently, GABA is considered to be a multifunctional molecule with various physiological effects throughout the body [9, 10]. In the brain, many researchers have found that the regulation of GABAR has preventive effects against OS-induced damage [5, 7, 8]. These results in the brain were mainly explained via specific pathways against OS (i.e., inhibition of the response to DNA damage [5, 11, 12] and promotion of cell survival [13, 14] or the free radical scavenging system [15, 16]). Liver contains GABA and its transporter [10], and hepatic GABAR has been also detected [17]. However, in the liver, the effects of GABAR regulation have not been reported.

Orthotopic liver transplantation (OLT) is an accepted therapy for children and adults with end-stage liver disease, and it currently provides long-term survival and quality lifestyle. However, cold ischemia during organ storage and subsequent reperfusion severely damage the transplanted liver [18]. During cold ischemic preservation, parenchymal cells swell and bleb [18], and then Kupffer and endothelial cells trigger ROS/RNS production after warm reperfusion [18]. This cold ischemia/warm reperfusion (CIWR) injury is still a major cause of morbidity and mortality after OLT due to primary graft dysfunction or a nonfunctioning graft [18]. Reperfusion not only triggers the liver regeneration cascade but also causes fatal damage in the liver graft due to OS [18, 19]. Currently, strategic procedures are required to improve liver tolerance against CIWR injury. Proactive strategies through pharmacological pretreatment to limit graft damage from CIWR injury have the advantage of excellent graft function after OLT.

A small-for-size graft (SFSG) is also an issue in deceased-donor liver transplantation (DDLT) and living-donor liver transplantation (LDLT). An SFSG is defined as a ratio of graft weight against standard liver volume <40% [20, 21]. An inevitable insufficiency of graft size cannot be avoided in LDLT or split orthotopic liver transplantation (SOLT) for DDLT. Shear stress not only triggers the liver regeneration cascade but also causes fatal damage in the SFSG by OS [22, 23]. An SFSG in LDLT or SOLT is accompanied by CIWR injury, as well as shear stress with portal hypertension. Therefore, SFSGs result in a higher mortality and morbidity after LDLT or SOLT. The choice of a left-side graft is preferred from the viewpoint of greater donor safety and expanded donor candidates in LDLT [20, 24]. Guaranteed SOLT with successful outcomes resolves a donor shortage in DDLT [24, 25]. Currently, the 40%-SFSG is a critical issue [24].

Our laboratory has focused on the effect of GABAR regulation on liver damage by using rodent models [26–28]. We failed to show beneficial effects in GABAR regulation *ex vivo* and in GABAR regulation by a specific antagonist [27, 28]. However, GABAR regulation *in vivo* by a specific agonist showed a subtle reduction in liver damage in a murine hepatectomy model involving shear stress with portal hypertension [27] and in a rat orthotopic liver transplantation model with a whole-liver graft involving CIWR injury [26]. Proactive strategies through pharmacological pretreatment to limit graft damage from CIWR injury and shear stress with portal hypertension have advantages for overcoming a current issue.

As a final goal of GABAR regulation in the liver, we investigated the strategic potential of graft pretreatment *in vivo* by a GABAR agonist in the rat SOLT model with a 40%-SFSG, and we examined the possible pathways involved.

## 2. Materials and Methods

### 2.1. Animals

Lewis rats (RT-1^*l*^) were purchased from Harlan Laboratories, Inc. (Indianapolis, IN, USA). Graft donors and recipients were 8–12-week-old rats (approximately 250 g). The experimental protocols were approved by the Ethical Committee of our institution (Mayo Clinic, Institutional Animal Care and Use Committee, no. A19609). Rats were cared for in accordance with the institutional guidelines for Animal Welfare based on The National Institutes of Health Guide for the Care and Use of Laboratory Animals.

### 2.2. Intravenous Injection of a GABAR Agonist

A dose of 43.56 nmol/g body weight of GABAR agonist (GABA_A_ receptor agonist, muscimol, 114.10 g/mol; 70015, Fluka, Sigma-Aldrich Co., St. Louis, MO, USA) was used [5]. Four hours before graft harvest, the donor rat intravenously received 1.0 mL of GABAR agonist into the penile vein. 

### 2.3. SOLT with 40%-SFSG and Postoperative Care

Comprehensive details of the surgical procedures for rat SOLT and postoperative care in our institution have been previously described [29, 30]. Briefly, the syngeneic graft had a cold ischemic time of 2 h at 4°C in normal Ringer's solution. The liver graft was washed twice by 10 mL of normal Ringer's solution, immediately after the graft harvest and before graft implantation. The 40%-SFSG was made by the left median and lateral segments at the back table [29, 30]. To avoid any irrelevant signaling, the hepatic artery was reconstructed by ultramicrosurgery in this study [29, 30]. Each rat was housed separately after surgery, and body temperature was maintained by a heating pad. Postoperative observation was performed every 30 min until 6 h after SOLT, and 1.0 mL of warm lactate Ringer's solution was routinely administered every 1 h until 6 h after SOLT. In this model, we previously demonstrated the importance of a shortened anhepatic phase and exclusion of unreliable samples based on autopsy findings [29, 30]. In this study, the anhepatic phase was maintained within 20 min in each SOLT, and no surgical complications were observed in each case at autopsy.

### 2.4. Study Design

Recipient rats were divided into three groups according to donor treatments and the recipient's surgery as follows: (i) saline (normal saline, 1.0 mL, i.v.) and laparotomy, (ii) saline (normal saline, 1.0 mL, i.v.) and SOLT with 40%-SFSG, and (iii) GABAR agonist (muscimol, 43.56 nmol (4.98 *μ*g)/g body weight, 1.0 mL, i.v.) and SOLT with 40%-SFSG.

First, a survival study was performed (*n* = 10 in each group). Cell signalings involving cell proliferation, differentiation, and apoptosis were investigated from the early postoperative period [18, 31–33], and subsequently, progressive necrosis was observed [18, 31–33]. Serum, plasma, and liver samples for histopathological/immunohistological assessment and western blotting analyses were then collected 6 h after SOLT (*n* = 5 in each group).

### 2.5. Biochemical Assay and Coagulation Profile

Aspartate aminotransferase (AST), alanine aminotransferase (ALT), and total bilirubin (T-Bil) levels, and the international normalized ratio of prothrombin time (PT-INR) were measured. Serum AST, ALT, and T-Bil levels were assessed (SGOT, SGPT, and total bilirubin reagent, respectively, Biotron, Hemet, CA, USA). The PT-INR in plasma was measured by the i-STAT System (Abbott, Princeton, NJ, USA).

### 2.6. Histopathological and Immunohistological Assessments

Liver tissue was fixed in 10% neutral-buffered formalin, embedded in paraffin, and sliced into 4 *μ*m sections. Morphological characteristics and graft injury scores were assessed after hematoxylin-eosin (HE) staining. The graft damage score (points) has previously been described elsewhere [30, 34, 35]. Scores were counted in 10 fields (×100) in each slide, and then these scores were averaged.

Induction of apoptosis was assessed by immunostaining of terminal deoxynucleotidyl transferase-mediated deoxyuridine triphosphate nick-end labeling (TUNEL) (ApopTag Peroxidase *in situ* Apoptosis Detection Kit, S7100, Chemicon International, Inc., Billerica, MA, USA) and cysteine aspartic acid protease (caspase) 3 (cleaved caspase-3 (Asp175) antibody, 9661S, Cell Signaling Technology, Inc., Danvers, MA, USA). TUNEL-positive nuclei were stained brown, and negative nuclei were counterstained light blue. Caspase-3-positive nuclei were stained brown, and negative nuclei were counterstained blue. Slides were scanned with an automated high-throughput scanning system (Scanscope XT, Aperio Technologies, Inc., Vista, CA, USA). To quantify the immunohistological findings, positively stained nuclei were counted by Aperio Imagescope software (Aperio Technologies, Inc.). All nuclei were classified into four color intensity levels, and the higher two levels were considered as positive. The ratio of positively stained nuclei to all nuclei was calculated, and the mean ratio per mm^2^ was determined.

### 2.7. Western Blotting Analysis

The primary antibodies for 4-hydroxynonenal (4-HNE) (4 hydroxynonenal antibody, ab46545, Abcam, Cambridge, MA, USA), ataxia-telangiectasia mutated kinase (ATM) (phospho-ATM/ATR substrate rabbit mAb, Cell Signaling Technology), phosphorylated histone H2AX (phospho-histone H2AX antibody, 2577, Cell Signaling Technology), phosphatidylinositol-3 kinase (PI3K) (phospho-PI3K p85/p55 antibody, 4228, Cell Signaling Technology), Akt (phospho-Akt rabbit mAb, 4058, Cell Signaling Technology), superoxide dismutase (SOD) 1 (Cu/Zn superoxide dismutase, LS-B2907, LifeSpan BioSciences, Seattle, WA, USA), SOD 2 (Mn superoxide dismutase, LS-C62194, LifeSpan BioSciences), and catalase (catalase, LS-B2554, LifeSpan BioSciences) were used. Liver samples were collected, homogenized, and centrifuged at high speed for 10 min at 4°C. The supernatant was then collected and used for bicinchoninic acid protein determination (BCA Protein Assay Reagent, Thermo Fisher Scientific, Rockford, IL, USA) and western blot analysis. Forty micrograms of protein were run on 4–20% Tris-glycine gels and transferred onto 0.45 *μ*m nitrocellulose membranes. The membranes were then blocked with 5% nonfat milk made up in a Tris-buffered saline solution. After blocking, the membranes were incubated at 4°C overnight with the primary antibody. The next day, the membranes were washed three times for 10 min with Tris-buffered saline solution and then incubated with the peroxidase-conjugated secondary antibody for 1 h, with shaking at room temperature. After incubation, the membranes were once again washed three times for 10 min with Tris-buffered saline solution and then developed using chemiluminescence. Glyceraldehyde-3-phosphate dehydrogenase (GAPDH) served as a control. Signals were quantified by using ImageQuant 5.0 software (Molecular Dynamics, Sunnyvale, CA, USA).

### 2.8. Statistical Analysis

The results are presented as mean ± standard deviation. The Student's *t*-test was used for the comparison of unpaired continuous variables between groups. Survival curves were constructed by the Kaplan-Meier method (log-rank test). Statistical calculations were performed using SPSS Software Version 16.0 (SPSS Inc., Chicago, IL, USA). A *P* value <0.05 was considered statistically significant.

## 3. Results

### 3.1. Survival Curves

**Figure 1:**
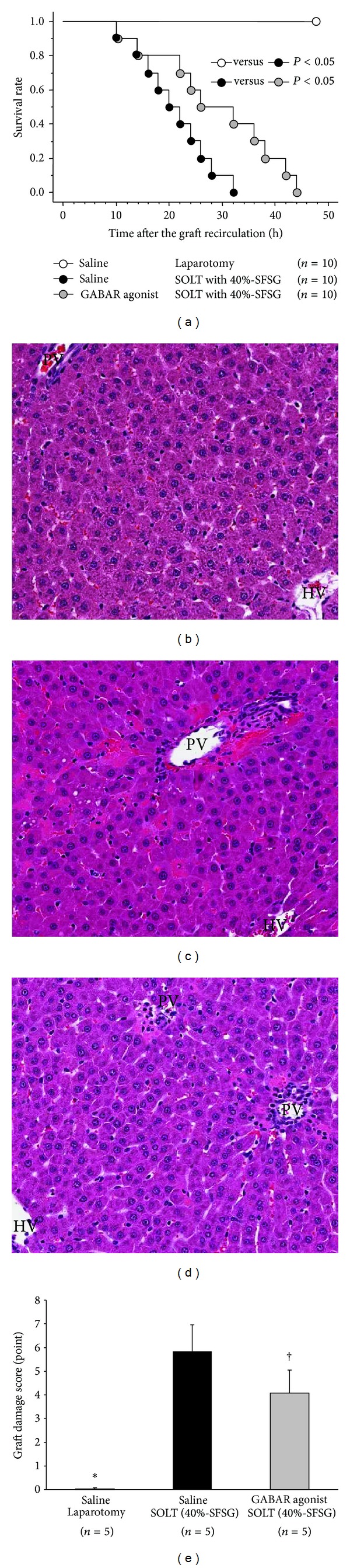
Survival curves, histopathological findings from HE staining, and graft damage scores. (a) Survival curves after SOLT with a 40%-SFSG. There were significant differences between laparotomy and SOLT with saline (*P* < 0.05*) and between SOLT with saline and SOLT with GABAR agonist (*P* < 0.05^†^). (b) Laparotomy with saline: H-E, ×100. (c) SOLT with saline: H-E, ×100. (d) SOLT with GABAR agonist: H-E, ×100. (e) Graft damage score: There were significant differences between laparotomy and SOLT with saline (*P* < 0.05*) and between SOLT with saline and SOLT with GABAR agonist (*P* < 0.05^†^). GABAR, *γ*-aminobutyric acid receptor; HE, hematoxylin-eosin; HV, hepatic vein; PV, portal vein; SFSG, small-for-size graft; and SOLT, split orthotopic liver transplantation.

Survival curves in each group are shown in Figure 1(a). SOLT with a 40%-SFSG clearly showed poorer survival than laparotomy (*P* < 0.0001), and graft pretreatment by GABAR agonist prolonged survival after SOLT (*P* = 0.0369).

### 3.2. Parenchymal Damage in Grafts

Inflammatory cell infiltration, vacuolization, hepatocyte ballooning, and necrosis were confirmed after SOLT with a 40%-SFSG. Actual histopathological findings in H-E staining are shown in each group in Figures 1(b)–1(d). 

There were significant differences between laparotomy and SOLT with saline (0.0 ± 0.0 versus 5.8 ± 1.1 points, *P* < 0.0001) and between SOLT with saline and SOLT with GABAR agonist (5.8 ± 1.1 versus 4.1 ± 1.0 points; *P* = 0.0280) (Figure 1(e)).

### 3.3. Biochemical and Coagulation Profiles

**Figure 2:**
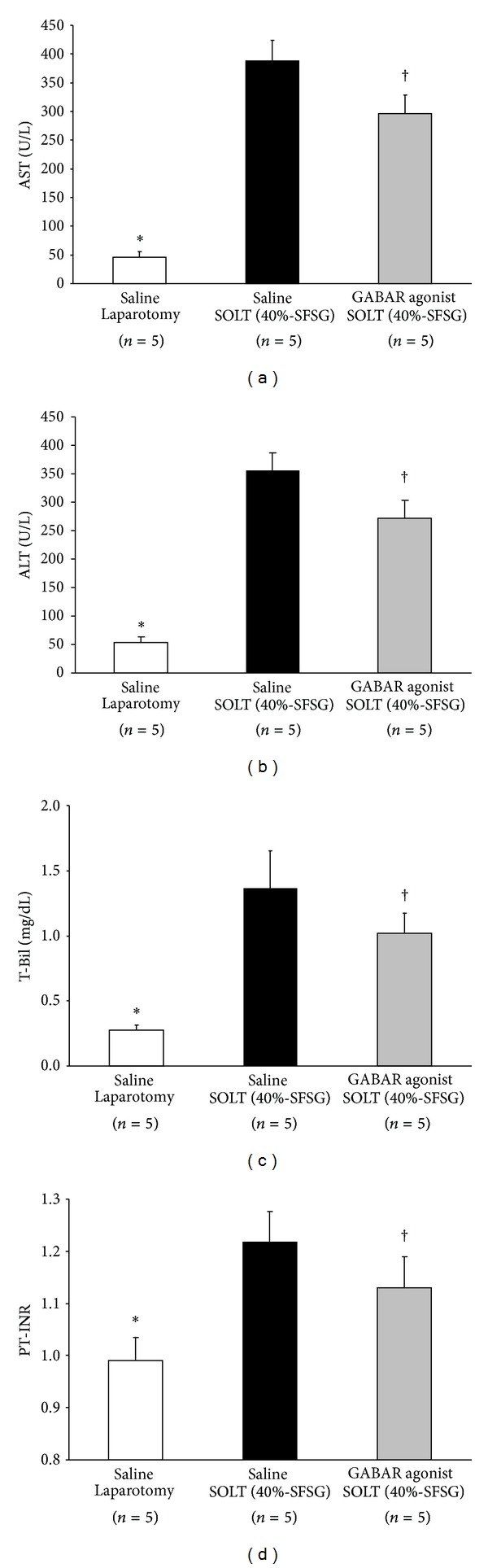
Biochemical and coagulation profiles. (a) AST levels. There were significant differences between laparotomy and SOLT with saline (*P* < 0.05*) and between SOLT with saline and SOLT with GABAR agonist (*P* < 0.05^†^). (b) ALT levels. There were significant differences between laparotomy and SOLT with saline (*P* < 0.05*) and between SOLT with saline and SOLT with GABAR agonist (*P* < 0.05^†^). (c) T-Bil levels. There were significant differences between laparotomy and SOLT with saline (*P* < 0.05*) and between SOLT with saline and SOLT with GABAR agonist (*P* < 0.05^†^). (d) PT-INR. There were significant differences between laparotomy and SOLT with saline (*P* < 0.05*) and between SOLT with saline and SOLT with GABAR agonist (*P* < 0.05†). AST, aspartate aminotransferase; ALT, alanine aminotransferase; GABAR, *γ*-aminobutyric acid receptor; SFSG, small-for-size graft; SOLT, split orthotopic liver transplantation; PT-INR, international normalized ratio of prothrombin time; and T-Bil, total bilirubin.

There were significant differences in serum AST levels between laparotomy and SOLT with saline (45.4 ± 10.3 versus 387.4 ± 36.8 U/L; *P* < 0.0001) and between SOLT with saline and SOLT with GABAR agonist (387.4 ± 36.8 versus 296.0 ± 32.3 U/L; *P* = 0.0031) (Figure 2(a)).

There were significant differences in serum ALT levels between laparotomy and SOLT with saline (54.2 ± 9.2 versus 354.2 ± 32.1 U/L; *P* < 0.0001) and between SOLT with saline and SOLT with GABAR agonist (354.2 ± 32.1 versus 272.4 ± 31.3 U/L; *P* = 0.0035) (Figure 2(b)).

There were significant differences in serum T-Bil levels between laparotomy and SOLT with saline (0.28 ± 0.04 versus 1.37 ± 0.29 mg/dL; *P* < 0.0001) and between SOLT with saline and SOLT with GABAR agonist (1.37 ± 0.29 versus 1.02 ± 0.15 mg/dL; *P* = 0.0453) (Figure 2(c)).

There were significant differences in PT-INR between laparotomy and SOLT with saline (0.99 ± 0.04 versus 1.22 ± 0.06; *P* = 0.0001) and between SOLT with saline and SOLT with GABAR agonist (1.22 ± 0.06 versus 1.13 ± 0.06; *P* = 0.0456) (Figure 2(d)).

### 3.4. Apoptotic Induction

**Figure 3:**
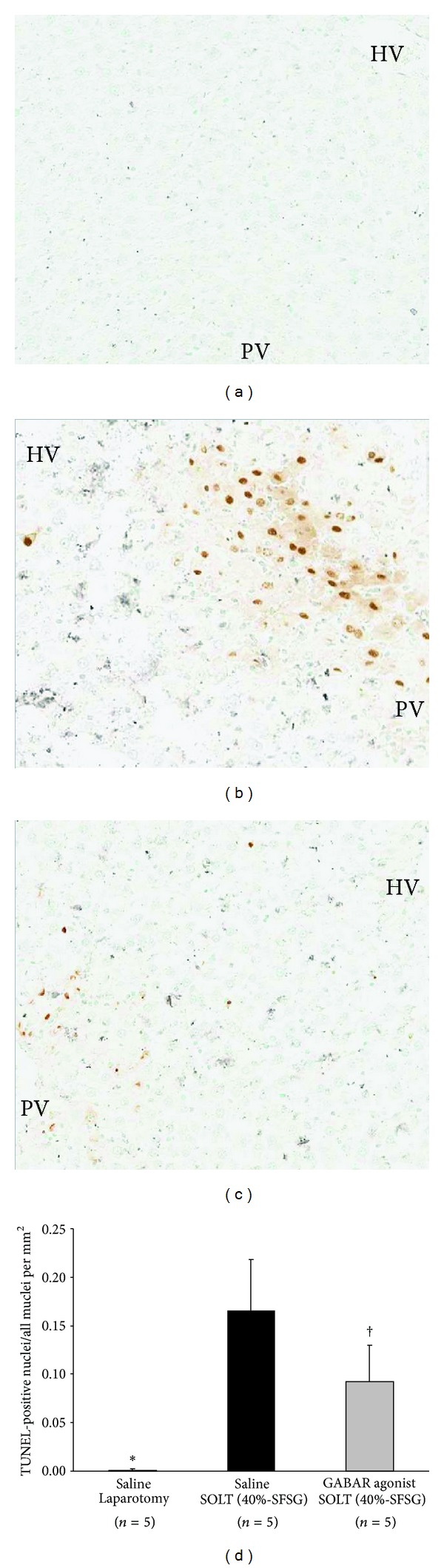
Immunohistological assessment by TUNEL. (a) Laparotomy with saline: TUNEL, ×100. (b) SOLT with saline: TUNEL, ×100. (c) SOLT with GABAR agonist: TUNEL, ×100. (d) Ratio of TUNEL-positive nuclei. There were significant differences between laparotomy and SOLT with saline (*P* < 0.05*) and between SOLT with saline and SOLT with GABAR agonist (*P* < 0.05^†^). GABAR, *γ*-aminobutyric acid receptor; HV, hepatic vein; SFSG, small-for-size graft; SOLT, split orthotopic liver transplantation; PV, portal vein; and TUNEL, terminal deoxynucleotidyl transferase-mediated deoxyuridine triphosphate nick-end labeling.

TUNEL immunostaining in each group is shown in Figures 3(a)–3(c). The ratio of TUNEL-positive nuclei was significantly different between laparotomy and SOLT with saline (0.001 ± 0.002 versus 0.166 ± 0.052; *P* < 0.0001) and between SOLT with saline and SOLT with GABAR agonist (0.166 ± 0.052 versus 0.092 ± 0.038; *P* = 0.0324) (Figure 3(d)).

**Figure 4:**
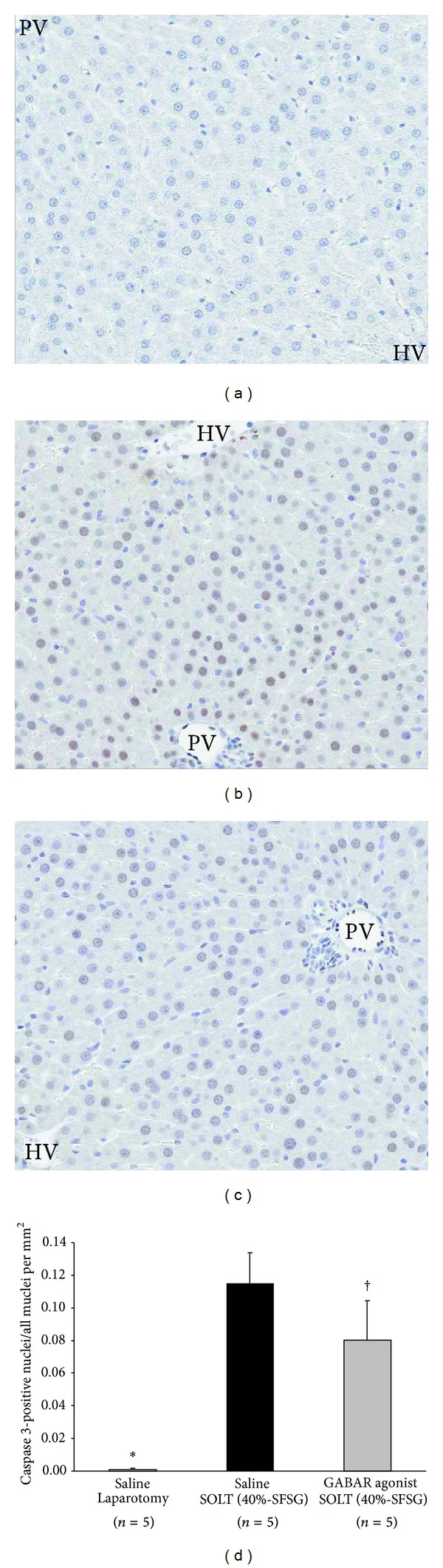
Immunohistological assessment by caspase 3. (a) Laparotomy with saline: caspase 3, ×100. (b) SOLT with saline: caspase 3, ×100. (c) SOLT with GABAR agonist: caspase 3, ×100. (d) The ratio of caspase 3-positive nuclei. There were significant differences between laparotomy and SOLT with saline (*P* < 0.05*) and between SOLT with saline and SOLT with GABAR agonist (*P* < 0.05^†^). Caspase, cysteine aspartic acid protease; GABAR, *γ*-aminobutyric acid receptor; HV, hepatic vein; SFSG, small-for-size graft; SOLT, split orthotopic liver transplantation; and PV, portal vein.

Caspase 3 immunostaining in each group is shown in Figures 4(a)–4(c). The ratio of caspase 3-positive nuclei was significantly different between laparotomy and SOLT with saline (0.0001 ± 0.0001 versus 0.115 ± 0.019; *P* < 0.0001) and between SOLT with saline and SOLT with GABAR agonist (0.115 ± 0.019 versus 0.080 ± 0.024; *P* = 0.0347) (Figure 4(d)).

### 3.5. Lipoperoxidation

**Figure 5:**
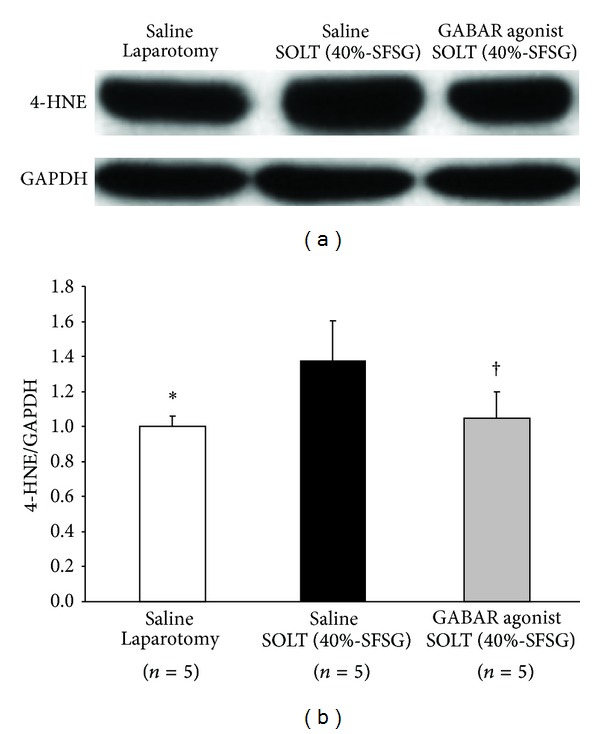
Western blot analysis of 4-HNE. (a) Intensities of 4-HNE and GAPDH. (b) Normalized 4-HNE. There were significant differences between laparotomy and SOLT with saline (*P* < 0.05*) and between SOLT with saline and SOLT with GABAR agonist (*P* < 0.05^†^). 4-HNE, 4-hydroxynonenal; GABAR, *γ*-aminobutyric acid receptor; GAPDH, glyceraldehyde-3-phosphate dehydrogenase; SFSG, small-for-size graft; and SOLT, split orthotopic liver transplantation.

Actual intensities of 4-HNE are shown in Figure 5(a). Normalized 4-HNE showed significant differences between laparotomy and SOLT with saline (1.00 ± 0.06 versus 1.38 ± 0.22; *P* = 0.0068) and between SOLT with saline and SOLT with GABAR agonist (1.38 ± 0.22 versus 1.05 ± 0.15; *P* = 0.0276) (Figure 5(b)).

### 3.6. Response to and Repair of DNA Damage

**Figure 6:**
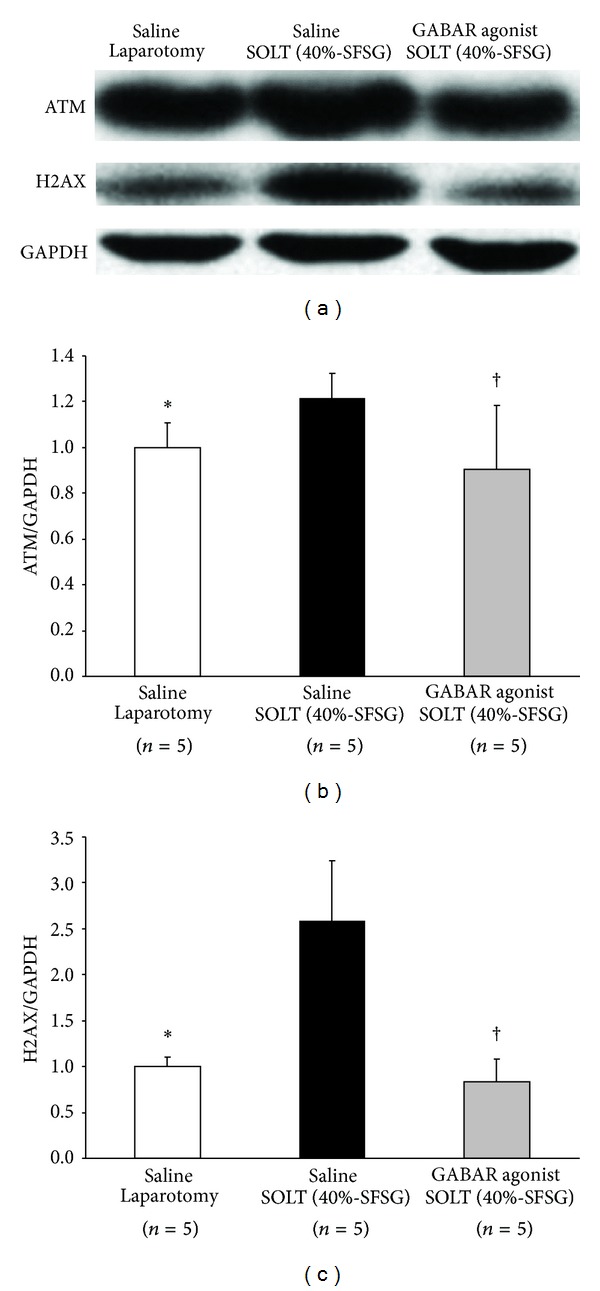
Western blot analyses of ATM and H2AX. (a) Intensities of ATM, H2AX, and GAPDH. (b) Normalized ATM. There were significant differences between laparotomy and SOLT with saline (*P* < 0.05*) and between SOLT with saline and SOLT with GABAR agonist (*P* < 0.05^†^). (c) Normalized H2AX. There were significant differences between laparotomy and SOLT with saline (*P* < 0.05*) and between SOLT with saline and SOLT with GABAR agonist (*P* < 0.05^†^). ATM, ataxia-telangiectasia mutated kinase; GABAR, *γ*-aminobutyric acid receptor; GAPDH, glyceraldehyde-3-phosphate dehydrogenase; SFSG, small-for-size graft; and SOLT, split orthotopic liver transplantation.

Actual intensities of ATM and H2AX in each group are shown in Figure 6(a).

Normalized ATM showed significant differences between laparotomy and SOLT with saline (1.00 ± 0.11 versus 1.21 ± 0.11; *P* = 0.0131) and between SOLT with saline and SOLT with GABAR agonist (1.21 ± 0.11 versus 0.90 ± 0.28; *P* = 0.0477) (Figure 6(b)). 

 Normalized H2AX showed significant differences between laparotomy and SOLT with saline (1.00 ± 0.10 versus 2.59 ± 0.66; *P* = 0.0007) and between SOLT with saline and SOLT with GABAR agonist (2.59 ± 0.66 versus 0.83 ± 0.25; *P* = 0.0005) (Figure 6(c)).

### 3.7. Promotion of Cell Survival

**Figure 7:**
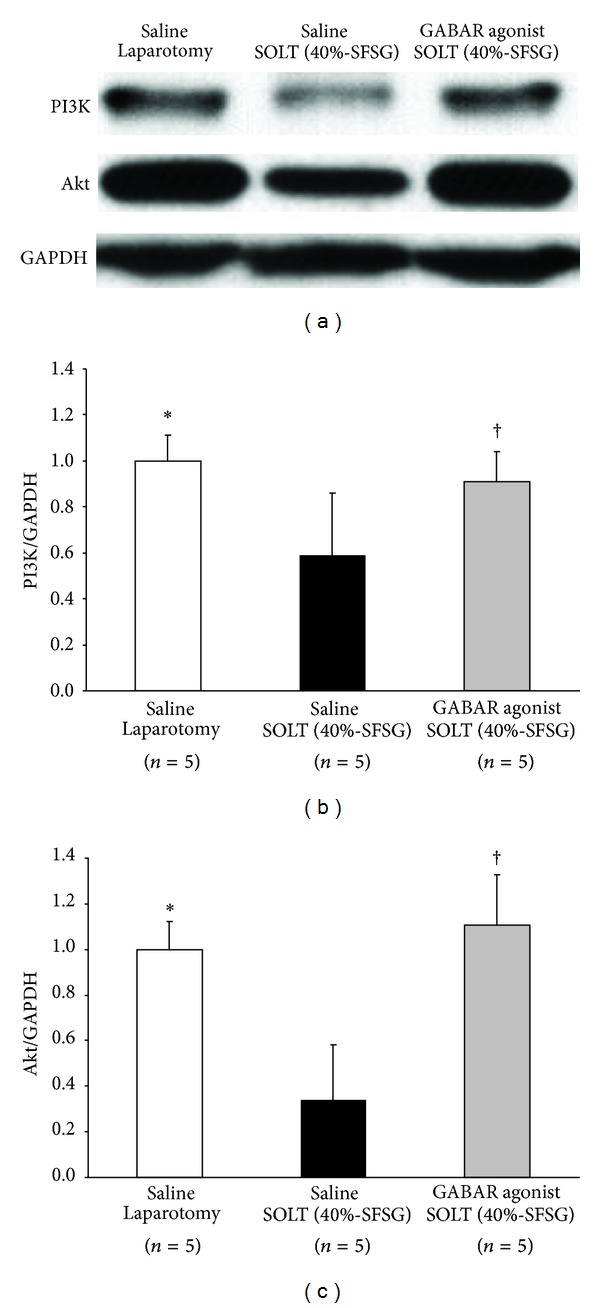
Western blot analyses of PI3K and Akt. (a) Intensities of PI3K. Akt, and GAPDH. (b) Normalized PI3K: There were significant differences between laparotomy and SOLT with saline (*P* < 0.05*) and between SOLT with saline and SOLT with GABAR agonist (*P* < 0.05^†^). (c) Normalized Akt. There were significant differences between laparotomy and SOLT with saline (*P* < 0.05*) and between SOLT with saline and SOLT with GABAR agonist (*P* < 0.05^†^). GABAR, *γ*-aminobutyric acid receptor; GAPDH, glyceraldehyde-3-phosphate dehydrogenase; PI3K, phosphatidylinositol-3 kinase; SFSG, small-for-size graft; and SOLT, split orthotopic liver transplantation.

Actual intensities of PI3K and Akt in each group are shown in Figure 7(a).

Normalized PI3K showed significant differences between laparotomy and SOLT with saline (1.00 ± 0.11 versus 0.59 ± 0.27; *P* = 0.0139) and between SOLT with saline and SOLT with GABAR agonist (0.59 ± 0.27 versus 0.92 ± 0.13; *P* = 0.0443) (Figure 7(b)). 

Normalized Akt showed significant differences between laparotomy and SOLT with saline (1.00 ± 0.12 versus 0.34 ± 0.24; *P* = 0.0006) and between SOLT with saline and SOLT with GABAR agonist (0.34 ± 0.24 versus 1.11 ± 0.22; *P* = 0.0007) (Figure 7(c)).

### 3.8. Activities of Antioxidant Enzymes

**Figure 8:**
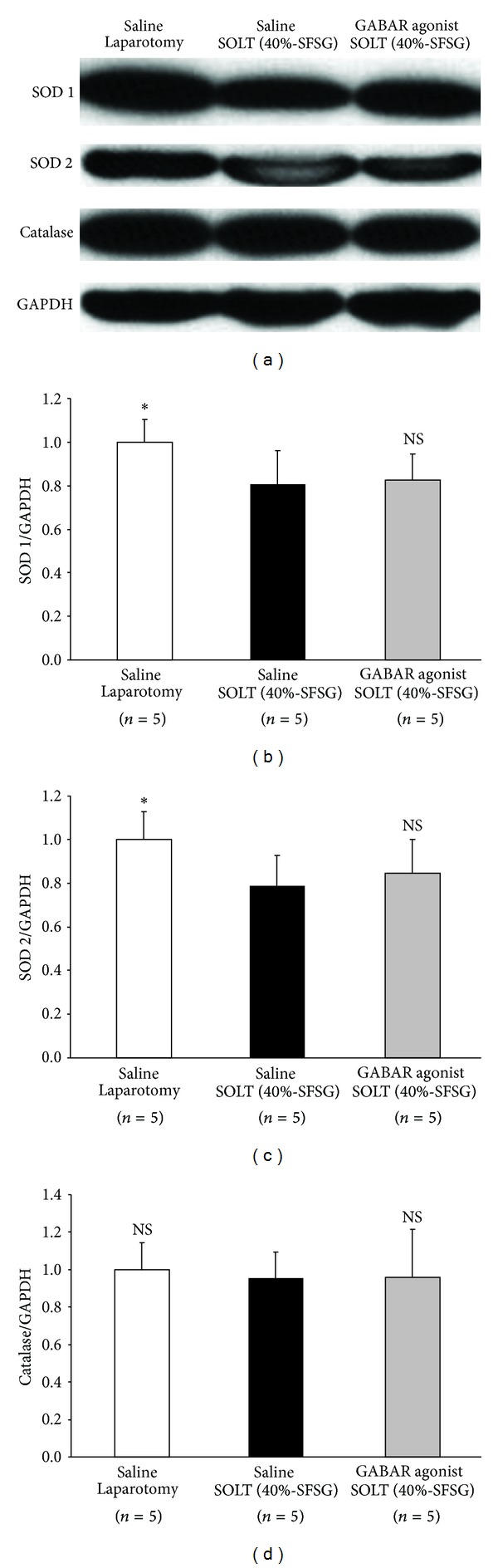
Western blot analyses of SOD 1, SOD 2, and catalase. (a) Intensities of SOD 1, SOD 2, catalase, and GAPDH. (b) Normalized SOD 1. There were significant differences between laparotomy and SOLT with saline (*P* < 0.05*) but no differences between SOLT with saline and SOLT with GABAR agonist. (c) Normalized SOD 2. There were significant differences between laparotomy and SOLT with saline (*P* < 0.05*) but no differences between SOLT with saline and SOLT with GABAR agonist. (d) Normalized catalase. There were no significant differences between laparotomy and SOLT with saline and between SOLT with saline and SOLT with GABAR agonist. GABAR, *γ*-aminobutyric acid receptor; GAPDH, glyceraldehyde-3-phosphate dehydrogenase; NS, not significant (*P* ≥ 0.05); SFSG, small-for-size graft; SOD, superoxide dismutase; SOLT, split orthotopic liver transplantation.

Actual intensities of SOD 1, SOD 2, and catalase in each group are shown in Figure 8(a).

Normalized SOD 1 showed significant differences between laparotomy and SOLT with saline (1.00 ± 0.10 versus 0.81 ± 0.16; *P* = 0.0445) but not between SOLT with saline and SOLT with GABAR agonist (0.81 ± 0.16 versus 0.82 ± 0.12; *P* = 0.8248) (Figure 8(b)).

Normalized SOD 2 showed significant differences between laparotomy and SOLT with saline (1.00 ± 0.13 versus 0.79 ± 0.14; *P* = 0.0361) but not between SOLT with saline and SOLT with GABAR agonist (0.79 ± 0.14 versus 0.84 ± 0.15; *P* = 0.5765) (Figure 8(c)).

Normalized catalase showed no significant differences between laparotomy and SOLT with saline (1.00 ± 0.14 versus 0.95 ± 0.14; *P* = 0.6904) and between SOLT with saline and SOLT with GABAR agonist (0.95 ± 0.14 versus 0.96 ± 0.26; *P* = 0.9764) (Figure 8(d)).

## 4. Discussion

 Based on the current situation in the clinical field, the 40%-SFSG needs to be investigated in detail because successful SOLT overcomes a donor shortage in DDLT, and the shift to a left-lobe graft provides donor safety in LDLT [20, 24, 30]. However, the 40%-SFSG is prone to ischemia/reperfusion injury and shear stress with portal hypertension, and therefore, the OS-induced damage after SOLT is more fatal [18, 36–38]. In our study, a survival study, biochemical assays, and histopathological assessment showed that the 40%-SFSG received the liver injury enough. OS causes DNA damage and subsequent apoptosis [1–3], and in our study, immunohistochemistry showed that SOLT induced apoptosis in the 40%-SFSG. ROS/RNS can attack and damage a variety of critical biological molecules [1–3], and the products of lipid peroxidation reliably and rapidly reflect sensitive and specific signals due to OS occurring *in vivo* [39, 40]. The fatty aldehyde 4-HNE is an end product of lipoperoxidation [39, 40]. Our results of 4-HNE showed that OS occurred after SOLT. Therefore, OS after SOLT with a 40%-SFSG resulted in apoptotic induction and subsequent necrosis.

OS mediated by free radicals is defined as an imbalance between the production of ROS/RNS and antioxidant capacity [1–3]. ROS/RNS have been suggested as a major contributing factor for DNA damage in the progression of OS. As a sensor of DNA damage responses, the protein kinase ATM can be initiated through rapid intermolecular autophosphorylation induced by DNA damage [12, 41]; it phosphorylates various proteins, and subsequently amplifies the responses to DNA damage [12]. This DNA damage-inducible kinase activates histone H2AX [5]. H2AX is required for cell cycle arrest and DNA repair following double-stranded DNA breaks [5, 42]. DNA damage results in the rapid phosphorylation of H2AX by ATM at sites of DNA damage [5, 43–45]. Our study showed that this response to and repair of DNA damage via ATM/H2AX was clearly triggered after SOLT with a 40%-SFSG and that this cascade is a possible pathway in the process of OS-induced injury after SOLT with SFSG. Our preliminary data in the rat OLT model with whole-liver grafts (i.e., a model for only CIWR injury) suggested that GABAR regulation by a specific agonist showed differences in ATM/H2AX [26]. We consider that GABAR regulation may have a beneficial effect against CIWR injury via the ATM/H2AX pathway in the liver.

From the viewpoint of the production of ROS/RNS in the process of OS, Akt also plays a critical role in controlling apoptosis [41, 46, 47] and promotes cell survival [47–50]. Apoptotic machinery is inhibited by the activation of Akt [46, 51, 52]. Akt is a component of the antiapoptotic process related to the activation of PI3K [14], and PI3K is upstream from Akt [47, 53]. The cell survival pathway via PI3K/Akt is also considered as an important signaling pathway to control apoptotic induction in the liver [54, 55]. Our study showed that this promotion of cell survival via PI3K/Akt was disturbed after SOLT with a 40%-SFSG and that this cascade could be one of the possible pathways in the process of OS-induced injury after SOLT with SFSG. Our preliminary data in the murine hepatectomy model (i.e., a model for only shear stress with portal hypertension) suggested that GABAR regulation by a specific agonist showed differences in PI3K/Akt [27]. Therefore, we consider that GABAR regulation may have a beneficial effect against shear stress with portal hypertension via the PI3K/Akt pathway in the liver.

From the viewpoint of antioxidant defenses, free radical scavenging enzymes, such as SOD and catalase, also play an important role in reducing DNA damage and subsequent apoptosis [2, 3, 56]. Normal cells are able to defend themselves against OS through this scavenging system [3, 56]. Our study showed a decrease in SOD 1 and SOD 2 levels after SOLT with a 40%-SFSG, although we initially expected that antioxidant enzymes would increase. Our results appear to be consistent with a previous opinion that OS impairs mitochondrial importing and processing of SOD [57]. However, another possible explanation for our results may be that this scavenging system failed, and some reactive molecules evaded the detoxification process and damaged potential targets because of drastic damage after SOLT with a 40%-SFSG, even though these scavenging enzymes can handle large amounts of ROS/RNS [58]. 

Our results of the survival study, histopathological assessment, and biochemical assays showed that pretreatment for SFSG by GABAR regulation *in vivo* affected graft damage after SOLT. Moreover, immunohistochemistry showed that this pretreatment reduced apoptotic induction after SOLT. In the field of brain research, the effect of GABAR regulation on the prevention of OS has been reported [5–7]. Although GABA was initially thought to be confined to the central nervous system, GABA is currently considered to be a multifunctional molecule with various physiological effects throughout the body [9, 10]. Although the liver contains GABA and hepatic GABAR [10, 17], the effects of GABAR regulation in the liver are unknown. Our study suggests that GABAR regulation may have a strategic potential for 40%-SFSGs as a pharmacological pretreatment for reducing OS-induced damage after SOLT, although SOLT with a 40%-SFSG involves fatal OS due to dual damage (i.e., CIWR injury and shear stress with portal hypertension).

Any pretreatment in a living donor violates ethical policy and spoils donor regulations. Whether GABAR regulation *ex vivo* (i.e., a procedure during organ storage) is more suitable for LDLT is unknown. Although our results showed the strategic potential of GABAR regulation *in vivo* as a pretreatment for liver grafts, we failed to confirm a positive effect of GABAR regulation *ex vivo* [28]. Therefore, some innovations are still required for clinical application.

In previous reports on the brain, many investigators have suggested that GABAR regulation by a specific agonist or antagonist affects the response to reduce OS-induced injury [5, 7, 8]. Their preventive effects in the brain have been mainly explained via specific pathways against OS (i.e., inhibition of the response to DNA damage [5, 11, 12] and promotion of cell survival [13, 14] or the free radical scavenging system [15, 16]). Many previous investigators have suggested that GABAR regulation in the brain has certain effects on the response to and repair of DNA damage via the ATM/H2AX pathway *in vivo* and *in vitro* in the process of OS [5, 11, 12]. Our study showed that the regulation of hepatic GABAR also appeared to reduce OS-induced DNA damage via the ATM/H2AX pathway as well as to have effects in the brain. With regard to the effects of GABAR regulation on OS in the brain, the PI3K/Akt pathway promotes cell survival against DNA damage [5, 13, 14, 46, 59]. Our study showed that regulation of hepatic GABAR appeared to promote cell survival via the PI3K/Akt pathway against OS-induced DNA damage as well as to have effects in the brain. However, antioxidant enzymes reduce OS-induced damage. From the viewpoint of this scavenging system, some researchers have shown that GABAR regulation in the brain has preventive effects against OS-induced damage via antioxidant enzymes [15, 16]. Although SOD 2 plays an important role in preventing DNA damage in the SFSG [36], our results suggested that the effects of the regulation of hepatic GABAR against OS did not depend on this scavenging system. Overall, we speculate that the regulation of hepatic GABAR has a preventive effect against OS, by reducing DNA damage via the ATM/H2AX pathway and by promoting cell survival via the PI3K/Akt pathway. However, antioxidant enzymes might be important for GABAR regulation in the brain [15, 16]. 

## 5. Conclusion

In conclusion, regulation of GABAR by a specific agonist *in vivo* works well in the liver, as well as the brain. Even though CIWR injury and shear stress with portal hypertension affect 40%-SFSGs after SOLT and results in fatal OS, graft pretreatment *in vivo* by GABAR regulation clearly improves graft damage after SOLT. This strategy may be advantageous for overcoming current issues in the DDLT and LDLT fields. The effects of GABAR regulation on graft damage after SOLT with a 40%-SFSG appear to prevent OS by reducing DNA damage via the ATM/H2AX pathway and by promoting cell survival via the PI3K/Akt pathway.
